# Supplementary material for: Impact of tiered restrictions on human activities and the epidemiology of the second wave of COVID-19 in Italy
Source: Nat Commun. 2021 Jul 27;12:4570. doi: 10.1038/s41467-021-24832-z (PMC8316570; doi:10.1038/s41467-021-24832-z)
Supplement: Supplementary file 1 — Supplementary Information [file 41467_2021_24832_MOESM1_ESM.pdf]

Supplementary Information

Impact of tiered restrictions on human activities and the epidemiology of the second wave of COVID-19 in Italy

Mattia Manica [1,2], Giorgio Guzzetta [1, 2], Flavia Riccardo [3], Antonio Valenti [4], Piero Poletti [1,2], Valentina Marziano [1,2], Filippo Trentini [1,2], Xanthi Andrianou [3,5], Alberto Mateo-Urdiales [3,6], Martina del Manso [3,6], Massimo Fabiani [3], Maria Fenicia Vescio [3], Matteo Spuri [3], Daniele Petrone [3], Antonino Bella [3], Sergio Iavicoli [4,#], Marco Ajelli [7,8,#], Silvio Brusaferro [3,#], Patrizio Pezzotti [3,#], Stefano Merler [1,2,#, *]

[1] Center for Health Emergencies, Bruno Kessler Foundation, Trento, Italy
[2] Epilab-JRU, FEM-FBK Joint Research Unit, Trento, Italy
[3] Istituto Superiore di Sanità, Rome, Italy
[4] Italian Workers' Compensation Authority (INAIL), Department of Occupational and Environmental Medicine, Epidemiology and Hygiene, Rome, Italy
[5] Cyprus University of Technology, Limassol, Cyprus
[6] European Programme for Intervention Epidemiology Training (EPIET), European Centre for Disease Prevention and Control (ECDC), Stockholm, Sweden
[7] Department of Epidemiology and Biostatistics, Indiana University School of Public Health, Bloomington, United States
[8] Laboratory for the Modeling of Biological and Socio-technical Systems, Northeastern University, Boston, United States
* corresponding author: Stefano Merler, merler@fbk.eu
[#] These authors jointly supervised this work

1. Changes in human activities after the introduction of tiers

Table S1 reports the results of the linear mixed models on the Google mobility data for each of the location category. Parameter β_0 represents the category's mean mobility before the introduction of the tiers, while parameters $\beta_1, \beta_2, \beta_3$ represent the difference in the mean mobility for provinces in yellow, orange and red tiers respectively, compared to β_0. p-values for t-statistics were computed using Satterthwaite's method for denominator degrees of freedom considering statistical significance at the 0.05 threshold (p-values <0.05)."Reported values represent percentage points compared to the pre-pandemic baseline.

Table S1 Results of linear mixed models on Google mobility data for each location.

Location	Parameter	Value	Std Error	DF	t-value	p-value
**Grocery/ Pharmacy**	β_0	-0.89925	0.917075	-0.98056	15.94459	0.341454
	β_1	-5.25475	0.48196	-10.9029	164.3782	3.59E-21
	β_2	-11.2807	0.517082	-21.8162	164.9524	1.75E-50
	β_3	-21.1227	0.562332	-37.5628	165.8015	5.29E-83
**Parks**	β_0	11.09021	1.953271	5.677763	15.82006	3.58E-05
	β_1	-6.12371	1.433472	-4.27194	166.9954	3.25E-05
	β_2	-31.1464	1.536525	-20.2707	167.9877	5.38E-47
	β_3	-45.2031	1.668026	-27.0997	169.4064	1.74E-63
**Retail/ Recreation**	β_0	-20.8301	1.335306	-15.5995	14.69864	1.50E-10
	β_1	-8.9815	0.534012	-16.8189	165.9061	1.10E-37
	β_2	-25.7174	0.573161	-44.8693	166.2494	8.09E-95
	β_3	-34.2708	0.623707	-54.9469	166.7844	9.32E-109
**Transit stations**	β_0	-19.6636	1.511468	-13.0096	16.93269	3.05E-10
	β_1	-14.725	0.6692	-22.0039	160.1254	2.83E-50
	β_2	-24.9473	0.718081	-34.7416	160.5276	1.41E-76
	β_3	-31.2401	0.787786	-39.6556	161.8096	2.98E-85
**Workplaces**	β_0	-16.6515	0.655163	-25.4159	19.38602	2.37E-16
	β_1	-6.74687	0.302839	-22.2787	163.3531	2.20E-51
	β_2	-11.5679	0.324983	-35.5954	163.7966	5.38E-79
	β_3	-15.9586	0.353539	-45.1396	164.4871	1.40E-94
**Residential**	β_0	6.85952	0.237311	28.90514	21.68241	8.33E-19
	β_1	3.709299	0.134483	27.58182	164.7453	1.23E-63
	β_2	7.841163	0.14426	54.35424	165.4399	1.97E-107
	β_3	10.07891	0.156845	64.26042	166.4686	1.98E-119

Figure S1 shows how the introduction of the tier system affected the mobility. Stricter tiers had a greater impact on mobility, as shown by increasing absolute values for the corresponding β parameters.

Figure S1. Distributions of changes in the time spent in different locations relative to pre-pandemic values, aggregated by tier (data at the province level). Boxplots represent the interquartile range (bounds of box), the 2.5% quantile (lower whisker), the 97.5% quantile (higher whisker) and the median (horizontal bar within the box) of the distributions. National interventions refer to the period October 14 – November 5, while mobility indicators in the tiers were computed over the period November 6 – November 25, n=107 observations (provinces).

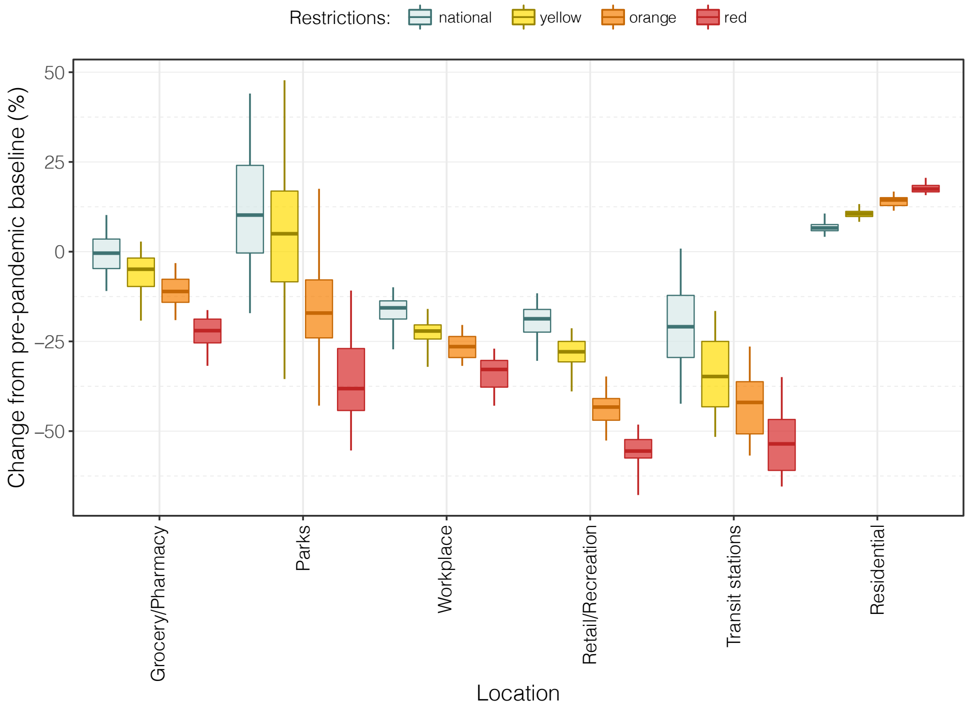

Restrictions: national yellow orange red
Change from pre-pandemic baseline (%)
50
25
0
−25
−50
Grocery/Pharmacy
Parks
Workplace
Retail/Recreation
Transit stations
Residential
Location

2. Changes in transmissibility

2.1 Additional details on models for the main analysis

Figure S2 shows the aggregate estimate of R(t), before and after the introduction of tiered restrictions at provincial scale. Figure S2 reports the temporal change observed in the estimated net reproduction number R(t) across different provinces of Italy, after the introduction of regional tiered restrictions.

Figure S2 Distribution of the estimated reproduction numbers R(t) across provinces aggregated according to tier and period of observation. Boxplots represent the interquartile range (bounds of box), the 2.5% quantile (lower whisker), the 97.5% quantile (higher whisker) and the median (horizontal bar within the box) of the R(t) distributions, n=107 observations (provinces) observed over the 2 time periods.

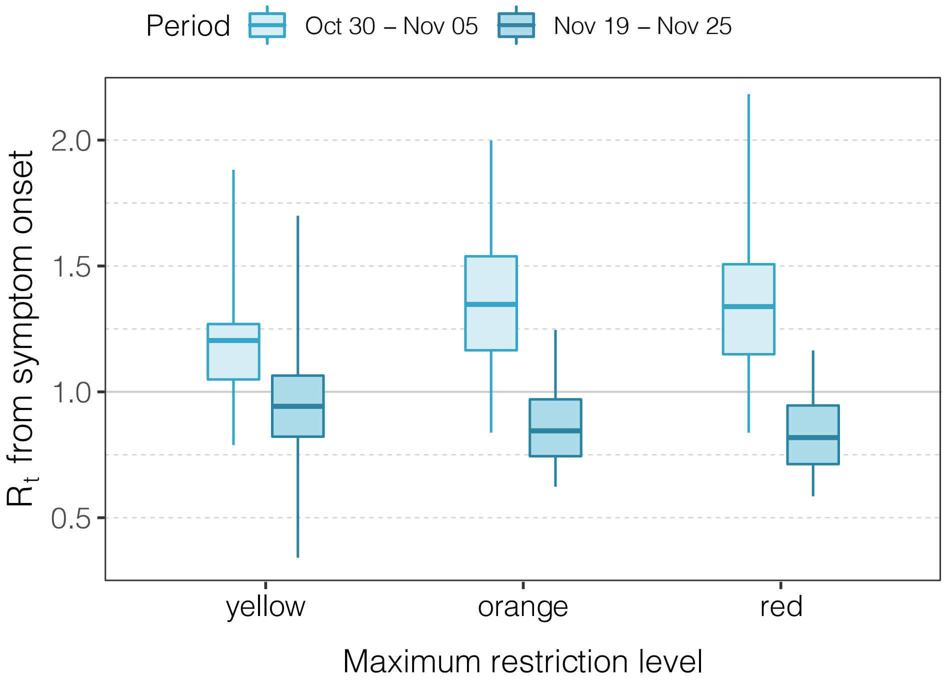

Figure S3 Variation of the observed net reproduction number R(t) in each province. The arrows indicate the variation in R(t) from the week before the introduction of regional tiered restrictions (October 30-November 5) to the end of our observations (November 19 – 25). Provinces are ordered by decreasing reduction in R(t).

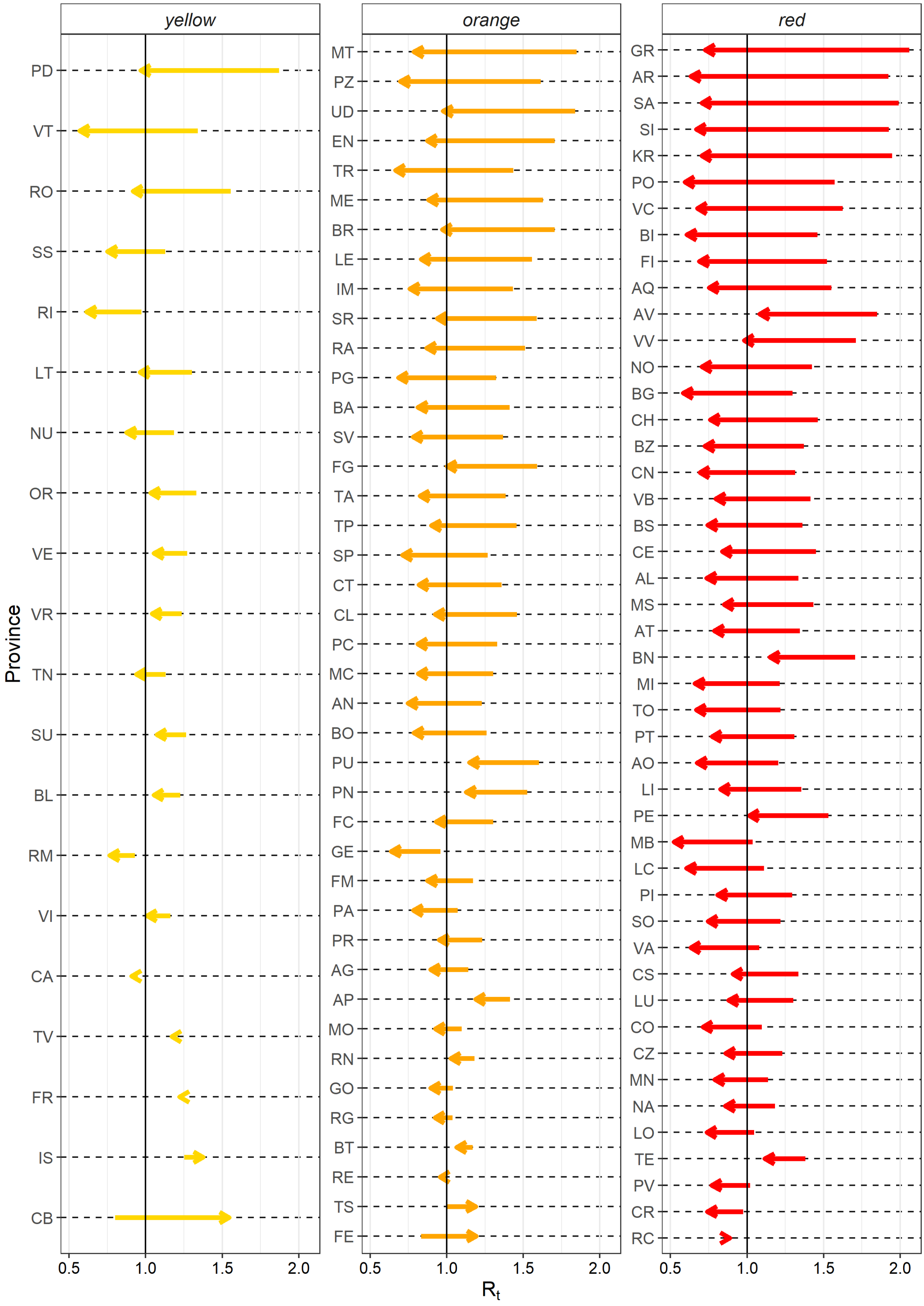

The association between tiers and changes in transmissibility was explored using six alternative models, which are fully described hereafter.

Model A

We applied a linear mixed model on the estimated SARS-CoV-2 transmissibility (i.e., the mean value of R(t)) represented by the following equation:

$$Y_{p,T} = \beta_0 + \beta_1 X_p^{orange} + \beta_2 X_p^{red} + \beta_3 Z_T + \beta_4 X_p^{orange} Z_T + \beta_5 X_p^{red} Z_T + a_r + b_{r,p} + \varepsilon_{p,T}$$

where

- $Y_{p,T}$ represents the mean value of R(t) in each of the 107 Italian provinces (*p*), averaged over two possible time periods (*T*): October 30 to November 5 (i.e., when nationwide interventions were still in place) or November 19 to November 25 (i.e., two to three weeks after the introduction of the tier system).
- X_p^l is a binary variable set to 1 if province *p* belongs to a region with maximum assigned tier l, and 0 otherwise;
- Z_T is a binary variable set to 0 if T=October 30 – November 5 and to 1 if T=November 19 – November 25;
- $\beta_0, \beta_1, \beta_2, \beta_3, \beta_4$, and β_5 are model parameters, with β_0 representing the average value of R(t) during the period October 30 – November 5 for provinces with maximum tier yellow;
- a_r and $b_{r,p}$ are random effects, assumed to be normally distributed: a_r allows random deviations from the mean R(t) among regions, $b_{r,p}$ allows random deviations from the regional mean R(t) among provinces within a region;
- $\varepsilon_{p,T}$ is random noise assumed to be normally distributed.

Table S2. Results of the linear mixed model on the net reproduction number R(t) at provincial level (Model A). p-values for t-statistics were computed using Satterthwaite's method for denominator degrees of freedom considering statistical significance at the 0.05 threshold (p-values <0.05). The estimated standard deviation for the random effect between regions, a_r, was 0.09, while the one for the random effect between provinces of the same region, $b_{p,r}$ was $7 \cdot 10^{-10}$. The estimated standard deviation for random noise, $\varepsilon_{p,T}$, was 0.20. The estimated marginal and conditional coefficient of determination R^2 computed based on Nakagawa et al [1] were 0.580 and 0.648.

PARAMETER	INTERPRETATION	VALUE	STD ERROR	DF	T-VALUE	P-VALUE
$\boldsymbol{\beta_0}$	Mean R(t) before the introduction of tiers for provinces in maximum tier yellow	1.215	0.061	37.8	18.830	<0.00001
$\boldsymbol{\beta_1}$	Difference in the mean R(t) before the introduction of tiers for provinces in maximum tier orange, compared to yellow	0.138	0.076	35.3	1.821	0.07708
$\boldsymbol{\beta_2}$	Difference in the mean R(t) before the introduction of tiers for provinces in maximum tier red, compared to yellow	0.198	0.076	33.6	2.608	0.01348
$\boldsymbol{\beta_3}$	Reduction in R(t) for provinces in maximum tier yellow	-0.224	0.063	193.2	-3.559	0.00047
$\boldsymbol{\beta_4}$	Additional reduction in R(t) for provinces in maximum tier orange, on top of reduction afforded by yellow	-0.235	0.077	193.2	-3.063	0.00251

β_5	Additional reduction in R(t) for provinces in maximum tier red, on top of reduction afforded by yellow	-0.403	0.076	193.2	-5.341	<0.00001

Table S3 Net reproduction number R(t) before and after the tiered restrictions at provincial scale.

Maximum tier	Mean R(t) (95% CI) October 30 – November 5	Mean R(t) (95% CI) November 19 - 25	Relative reduction (95% CI)
**Yellow**	1.21 (1.09 – 1.33)	0.99 (0.87 – 1.11)	18.5% (9.0 – 27.5%)
**Orange**	1.35 (1.27 – 1.44)	0.89 (0.81 – 0.98)	34.0% (28.3 – 39.4%)
**Red**	1.41 (1.32 – 1.50)	0.79 (0.70 – 0.87)	44.3% (39.1 – 49.2%)

Residuals obtained from this analysis are reported in Figure S4.

Figure S4. Analysis of residuals for the linear model (main analysis). Left: distribution of Pearson residuals (i.e., raw residuals normalized with respect to the variance of residuals); right: scatterplot between Pearson residuals and fitted values of $Y_{p,T}$.

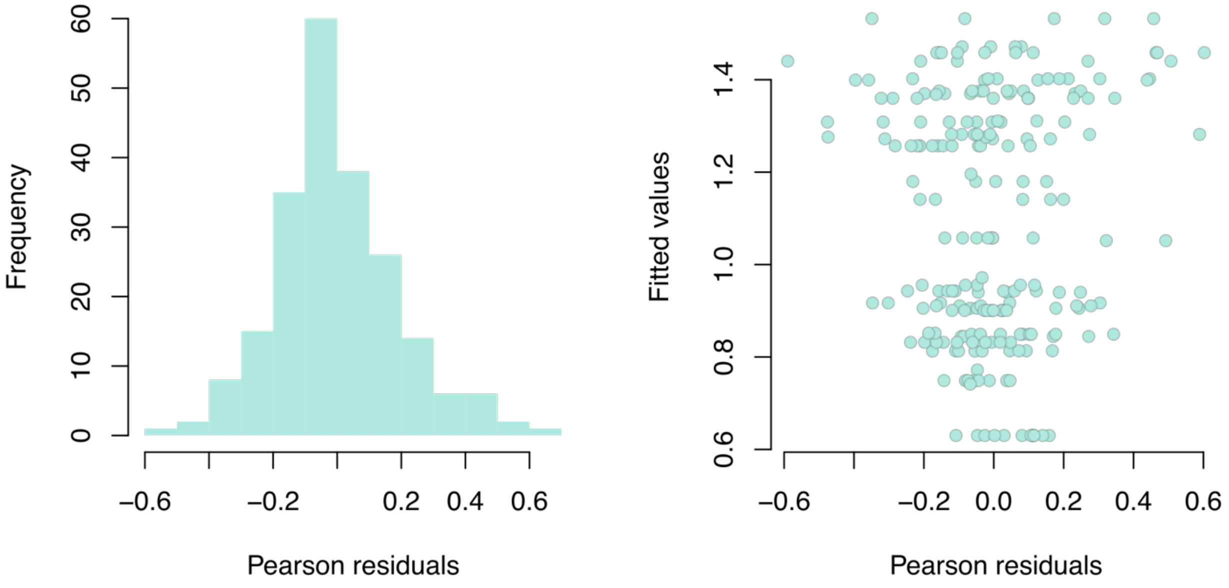

Model B

We applied a linear mixed model on R(t) represented by the following equation:

$$Y_{p,T} = \beta_0 + \beta_1 X_p^{orange} + \beta_2 X_p^{red} + \beta_3 Z_T + \beta_4 X_p^{orange} Z_T + \beta_5 X_p^{red} Z_T + a_r + b_{r,p} + \varepsilon_{p,T}$$

where

- $Y_{p,T}$ represents the log-transformed mean value of R(t) in each of the 107 Italian provinces (p), averaged over two possible time periods (T): October 30 to November 5 (i.e., when nationwide interventions were still in place) or November 19 to November 25 (i.e., two to three weeks after the introduction of the tier system).
- X_p^l is a binary variable set to 1 if province p belongs to a region with maximum assigned tier l, and 0 otherwise;
- Z_T is a binary variable set to 0 if T=October 30 – November 5 and to 1 if T=November 19 – November 25;
- $\beta_0, \beta_1, \beta_2, \beta_3, \beta_4$, and β_5 are model parameters, with β_0 representing the average value of the log-transformed R(t) during the period October 30 – November 5 for provinces with maximum tier yellow;
- a_r and $b_{r,p}$ are random effects, assumed to be normally distributed: a_r allows random deviations from the mean log-transformed R(t) among regions, $b_{r,p}$ allows random deviations from the regional mean log-transformed R(t) among provinces within a region;
- $\varepsilon_{p,T}$ is random noise assumed to be normally distributed.

Table S4. Results of the linear mixed model on the log-transformed reproduction number R(t) at provincial level (Model B). p-values for t-statistics were computed using Satterthwaite's method for denominator degrees of freedom considering statistical significance at the 0.05 threshold (p-values <0.05). The estimated standard deviation for the random effect between regions, a_r, was 0.08, while the one for the random effect between provinces of the same region, $b_{p,r}$ was $7 \cdot 10^{-10}$. The estimated standard deviation for random noise, $\varepsilon_{p,T}$, was 0.17. The estimated marginal and conditional coefficient of determination R^2 computed based on Nakagawa et al. [1] were 0.615 and 0.686, respectively.

PARAMETER	INTERPRETATION	VALUE	STD ERROR	DF	T-VALUE	P-VALUE
$\boldsymbol{\beta_0}$	Mean R(t) before the introduction of tiers for provinces in maximum tier yellow	0.179	0.054	35.4	3.291	0.00226
$\boldsymbol{\beta_1}$	Difference in the mean R(t) before the introduction of tiers for provinces in maximum tier orange, compared to yellow	0.102	0.067	33.1	1.520	0.13813
$\boldsymbol{\beta_2}$	Difference in the mean R(t) before the introduction of tiers for provinces in maximum tier red, compared to yellow	0.152	0.067	31.7	2.262	0.03070
$\boldsymbol{\beta_3}$	Reduction in R(t) for provinces in maximum tier yellow	-0.214	0.054	192.7	-3.983	<0.00001
$\boldsymbol{\beta_4}$	Additional reduction in R(t) for provinces in maximum tier orange, on top of reduction afforded by yellow	-0.197	0.066	192.7	-3.008	0.00298

$\boldsymbol{\beta_5}$	Additional reduction in R(t) for provinces in maximum tier red, on top of reduction afforded by yellow	-0.377	0.064	192.7	-5.857	<0.00001

Table S5 Net reproduction number Rt before and after the tiered restrictions at provincial scale.

Maximum tier	Mean Rt (95% CI) October 30 – November 5	Mean Rt (95% CI) November 19 - 25	Relative reduction (95% CI)
**Yellow**	1.20 (1.07 – 1.33)	0.97 (0.87 – 1.07)	19.0% (10.3 – 26.9%)
**Orange**	1.32 (1.22 – 1.43)	0.88 (0.81 – 0.95)	33.6% (28.4 – 38.1%)
**Red**	1.39 (1.29 – 1.51)	0.77 (0.71 – 0.83)	44.6% (40.5 – 48.3%)

Model C

We applied the same regression as in model A to R(t) values computed at provincial level on the subset of provinces where the same tier was constantly enacted over the whole study period. Specifically, we considered (see Figure 2 in the main text for a reference of category assignments):

- tier constantly yellow (20 provinces): Lazio (5 provinces), Molise (2 provinces), Sardinia (5 provinces), Trento (1 Autonomous Province), Veneto (7 provinces);
- tier constantly orange (15 provinces): Apulia (6 provinces), Sicily (9 provinces)
- tier constantly red (26 provinces): Aosta Valley (1 province), Calabria (5 provinces), Lombardy (12 provinces), Piedmont (8 provinces).

Table S6. Results of the linear mixed model on the net reproduction number R(t) at provincial level (Model C). p-values for t-statistics were computed using Satterthwaite's method for denominator degrees of freedom considering statistical significance at the 0.05 threshold (p-values <0.05). The estimated standard deviation for the random effect between regions, a_r, was 0.08, while the one for the random effect between provinces of the same region, $b_{p,r}$ was $7 \cdot 10^{-10}$. The estimated standard deviation for random noise, $\varepsilon_{p,T}$, was 0.18. The estimated marginal and conditional coefficient of determination R^2 computed based on Nakagawa et al. [1] were 0.580 and 0.651, respectively.

PARAMETER	INTERPRETATION	VALUE	STD ERROR	DF	T-VALUE	P-VALUE
$\boldsymbol{\beta_0}$	Mean R(t) before the introduction of tiers for provinces constantly in tier yellow	1.215	0.057	16.2	21.205	<0.00001
$\boldsymbol{\beta_1}$	Difference in the mean R(t) before the introduction of tiers for provinces constantly in tier orange, compared to yellow	0.208	0.095	11.4	2.188	0.050337
$\boldsymbol{\beta_2}$	Difference in the mean R(t) before the introduction of tiers for provinces constantly in tier red, compared to yellow	0.07	0.081	13.0	0.887	0.391458
$\boldsymbol{\beta_3}$	Reduction in R(t) for provinces constantly in tier yellow	-0.224	0.058	109.2	-3.855	0.000196
$\boldsymbol{\beta_4}$	Additional reduction in R(t) for provinces constantly in tier orange, on top of reduction afforded by yellow	-0.298	0.089	109.3	-3.357	0.001085
$\boldsymbol{\beta_5}$	Additional reduction in R(t) for provinces constantly in tier red, on top of reduction afforded by yellow	-0.318	0.077	109.3	-4.116	<0.00001

Table S7 Net reproduction number Rt before and after the tiered restrictions at provincial scale.

Maximum tier	Mean Rt (95% CI) October 30 – November 5	Mean Rt (95% CI) November 19 - 25	Relative reduction (95% CI)
**Yellow**	1.21 (1.10 – 1.33)	0.99 (0.88 – 1.10)	18.3% (9.4 – 27.3%)
**Orange**	1.42 (1.27 – 1.57)	0.90 (0.75 – 1.05)	36.7% (28.6 – 44.8%)
**Red**	1.29 (1.17 – 1.40)	0.74 (0.63 – 0.86)	42.4% (35.4 – 49.2%)

Model D

We applied model A to region-specific rather than province-specific data as follows:

$$Y_{r,T} = \beta_0 + \beta_1 X_r^{orange} + \beta_2 X_r^{red} + \beta_3 Z_T + \beta_4 X_r^{orange} Z_T + \beta_5 X_r^{red} Z_T + a_r + \varepsilon_{r,T}$$

where

- $Y_{p,T}$ represents the mean value of R(t) in each of the 21 Italian region and autonomous provinces (*r*), averaged over two possible time periods (*T*): October 30 to November 5 (i.e., when nationwide interventions were still in place) or November 19 to November 25 (i.e., two to three weeks after the introduction of the tier system).
- X_p^l is a binary variable set to 1 if *l* was the maximum tier enacted in the region *r*, and 0 otherwise;
- Z_T is a binary variable set to 0 if T=October 30 – November 5 and to 1 if T=November 19 – November 25;
- $\beta_0, \beta_1, \beta_2, \beta_3, \beta_4, \beta_5, \beta_6, \beta_7, \beta_8$ and β_9 are model parameters, with β_0 representing the average value of R(t) during the period October 30 – November 5 for region with maximum tier yellow;
- a_r is a random effect, assumed to be normally distributed: a_r allows random deviations from the mean R(t) among regions;
- $\varepsilon_{p,T}$ is random noise assumed to be normally distributed.

Table S8. Result of the linear mixed model on the net reproduction number R(t) at the regional level (Model D). p-values for t-statistics were computed using Satterthwaite's method for denominator degrees of freedom considering statistical significance at the 0.05 threshold (p-values <0.05). The estimated marginal and conditional coefficient of determination R^2 computed based on Nakagawa et al [1] were 0.701 and 0.714, respectively.

PARAMETERS	INTERPRETATION	VALUE	STD ERROR	DF	T-VALUE	P-VALUE
$\boldsymbol{\beta_0}$	Mean R(t) before the introduction of tiers for regions in maximum tier yellow	1.099	0.069	35.9	15.810	<0.00001
$\boldsymbol{\beta_1}$	Difference in the mean R(t) before the introduction of tiers for regions in maximum tier orange, compared to yellow	0.217	0.089	35.9	2.453	0.01914
$\boldsymbol{\beta_2}$	Difference in the mean R(t) before the introduction of tiers for regions in maximum tier red, compared to yellow	0.211	0.089	35.9	2.382	0.02265
$\boldsymbol{\beta_3}$	Reduction in R(t) for regions in maximum tier yellow	-0.087	0.096	18	-0.909	0.37562
$\boldsymbol{\beta_4}$	Additional reduction in R(t) for regions in maximum tier orange, on top of reduction afforded by yellow	-0.415	0.123	18	-3.389	0.00327
$\boldsymbol{\beta_5}$	Additional reduction in R(t) for regions in maximum tier red, on top of reduction afforded by yellow	-0.478	0.123	18	-3.901	0.00105

Table S9. Mean net reproduction number estimated from symptom onset, R(t), at regional level before and after regional interventions.

Maximum tier	Mean R(t) (95% CI) October 30 – November 5	Mean R(t) (95% CI) November 19 - 25	Relative reduction (95% CI)
**Yellow**	1.10 (0.96 – 1.24)	1.01 (0.88 – 1,15)	7.3% (-9.9 – 23.9%)
**Orange**	1.32 (1.21 – 1.42)	0.81 (0.71 – 0.92)	38.1% (27.5 – 47.0%)
**Red**	1.31 (1.20 – 1.42)	0.74 (0.64 – 0.85)	43.2% (33.8 – 52.2%)

Model E

It has been shown that the serial interval can be shortened by up to two-thirds when intensive contact tracing interventions are performed [2]. To explore the potential effect of contact tracing on the serial interval, we used the same model described in model A with the difference that the R(t) values considered as dependent variable (*Y)* were computed assuming a different serial interval. In particular, we estimated that about 63% of infections between October 1, 2020, and January 15, 2021, remained undetected in Italy [3]. Therefore, we assumed that the serial interval could have been reduced to a maximum of two thirds [2] for at most 37% of the overall number of infections. The resulting distribution of the serial interval would arise from a mixture of two gamma distributions, the one used in the main analysis (shape = 1.87, rate = 0.28) with weight 0.63, and the one representing the shortened serial interval (shape = 1.08, rate = 0.48, mean = 2.2 days) with weight 0.37. This mixture distribution was well approximated by a gamma with shape = 1.14 and rate = 0.23, therefore resulting in a mean serial interval of 5 days, i.e. about 25% shorter than the baseline (see Figure S5).

Figure S5. Density function of the serial interval distribution representing the reduction in the serial interval for notified cases. The blue line represents the serial interval distribution considered in model E as estimated from fitting the mixture (black dashed lines) of two gamma distributions: one having mean 2.2 days and weight 0.37 (red dashed lines), and one having mean 6.68 days and weight 0.63 (green dashed lines).

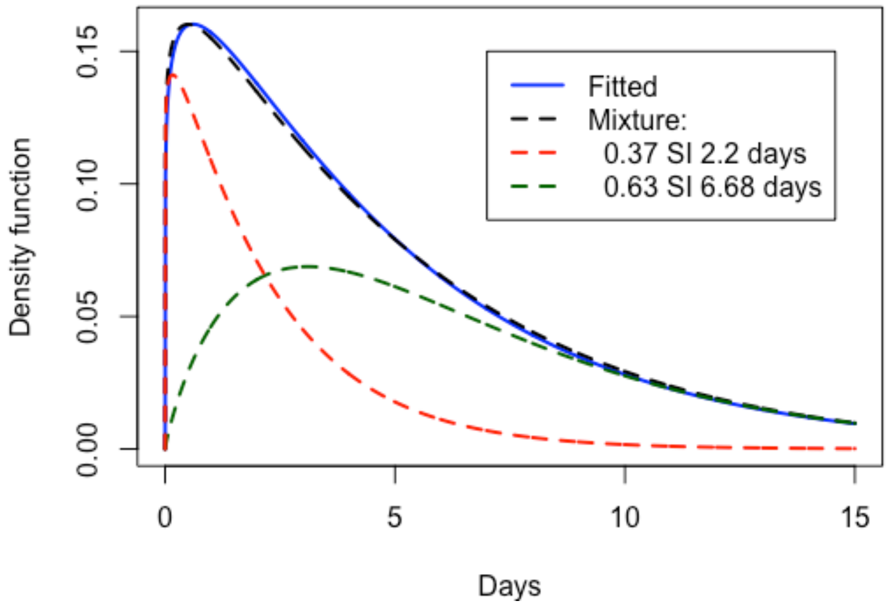

Table S10. Result of the linear mixed model on the net reproduction R(t) at the provincial level. See Table S2 for parameter interpretation. p-values for t-statistics were computed using Satterthwaite's method for denominator degrees of freedom considering statistical significance at the 0.05 threshold (p-values <0.05). The estimated marginal and conditional coefficient of determination R^2 computed based on Nakagawa et al [1] were 0.555 and 0.633, respectively.

PARAMETERS	VALUE	STD ERROR	DF	T-VALUE	P-VALUE
$\boldsymbol{\beta_0}$	1.177	0.049	32.6	24.023	<0.00001
$\boldsymbol{\beta_1}$	0.090	0.061	30.4	1.484	0.14844
$\boldsymbol{\beta_2}$	0.130	0.061	29.0	2.148	0.04021
$\boldsymbol{\beta_3}$	-0.152	0.049	190.9	-3.098	0.00224
$\boldsymbol{\beta_4}$	-0.187	0.060	190.9	-3.122	0.00207
$\boldsymbol{\beta_5}$	-0.322	0.059	190.9	-5.455	<0.00001

Table S11. Mean net reproduction number estimated from symptom onset, Rt, at regional level before and after regional interventions.

Maximum tier	Mean Rt (95% CI) October 30 – November 5	Mean Rt (95% CI) November 19 - 25	Relative reduction (95% CI)
**Yellow**	1.18 (1.08 – 1.27)	1.02 (0.93 – 1.12)	12.8% (4.9 – 20.5%)
**Orange**	1.27 (1.20 – 1.34)	0.93 (0.86 – 1.00)	26.7% (22.0 – 31.6%)
**Red**	1.31 (1.24 – 1.38)	0.83 (0.76 – 0.90)	36.3% (31.9 – 41.2%)

Model F

We estimated the potential impact of tiers by explicitly simulating the daily reduction of R(t) expected during each specific day spent in each tier at the regional scale. This approach allows to avoid the assignment of regions to a specific tier and to factor in the cumulative effect of changes in tier assignment over time. The model projects the daily number of hospitalized cases, using the renewal equation [4]:

$$H_r(t) = \mathrm{Pois}\left(\tilde{R}_r(t)\sum_{s=1}^{t}\varphi(s)h_r(t-s)\right)$$

where

- $Pois(\lambda)$ is a Poisson sample with rate λ;
- $h_r(t)$ is the daily number of new hospital admissions in region *r*;
- $\varphi(s)$ is the distribution of the generation time discretized by day, as above;
- $\tilde{R}_\mathrm{r}(t)$ is the modeled reproduction number over time.

We used as input for $h_i(t)$ the curve of daily hospital admissions in each region until November 5, 2020 (i.e., the day before the enactment of the tiers) and projected hospital admissions from November 6 to 25.

To model $\tilde{R}_\mathrm{r}(t)$, we assumed that each tier L would take two weeks to bring the reproduction number to a tier-specific, region-independent final value, denoted as $R_{f,L}$. Therefore, we defined a daily reduction in the reproduction number depending on the tier L and on the day of assignment of the tier, T, as $\delta_\mathrm{L}(T) = \frac{\tilde{R}_r(T)-R_{f,L}}{14}$. Then, $\tilde{R}_\mathrm{r}(t)$ was defined as:

$$\tilde{R}_\mathrm{r}(t \geq T) = \tilde{R}_r(T) - \delta_L(T)\cdot(t-T)$$

for t < 14 days or until a new reassignment occurs. If no reassignment occurs in the 14 days, $\tilde{R}_r(T)$ is then fixed at $R_{f,L}$, otherwise δ_L is recalculated depending on the time T' at which the new reassignment occurs and on the newly assigned tier, and the $\tilde{R}_\mathrm{r}(t \geq T')$ is defined as above. $\tilde{R}_r(0)$ is initialized by setting equal the average of the observed R(t) over the period 30 October – 5 November 2020 for the given region, and the first assignment of tiers occurs at T=0 (corresponding to November 5). For example, if a region starts from $\tilde{R}_r(0) = 2$ and is assigned a yellow tier at T=0, an orange tier at T=7 and a red tier at T=11, assuming that $R_{f,yellow} = 1$, $R_{f,orange} = 0.8$ and $R_{f,red} = 0.6$, then the $\tilde{R}_r(t)$ of this region will decrease linearly from 2 to 1.5 for 0 ≤ t ≤ 7, from 1.5 to 1.3 for 7 ≤ t ≤ 11 and from 1.3 to 0.6 for for 11 ≤ t ≤ 25, remaining at 0.6 after t = 25.

The three unknown values of $R_{f,L}$ were considered free model parameters and their posterior distributions were estimated by an MCMC approach, applying the Metropolis-Hastings sampling to a likelihood function defined as follows:

$$\mathcal{L} = \prod_{\mathrm{i}=1}^{\mathrm{Region}} \mathrm{P}\left(\mathrm{C(i)};\ \sum_{t=1}^{20} H_i(t))\right)$$

where

- P(k; λ) is the probability mass function of a Poisson distribution (i.e., the probability of observing k events if these events occur with rate λ);
- C(i) is the cumulative number of new cases observed in region *i* from 6 November (t=1) to

25 November (t=20), 2020

Summary results of the estimated posterior distributions are reported in Table S12 and a model validation is shown in Figure S6.

Table S12. Estimates of the final reproduction number associated to each tier after 14 days since its assignment, according to Model F.

Tier	Mean R(t) (95%CrI)
Yellow	0.950 (0.936 – 0.967)
Orange	0.804 (0.781 – 0.823)
Red	0.768 (0.757 – 0.780)

Figure S6 Validation of Model F. We compare the observed daily number of hospital admissions over the study period in each region to the corresponding model estimates. Dots represent the observed daily hospital admissions, solid lines represent the mean daily hospital admission simulated using model F, dashed lines represent their 95% confidence intervals.

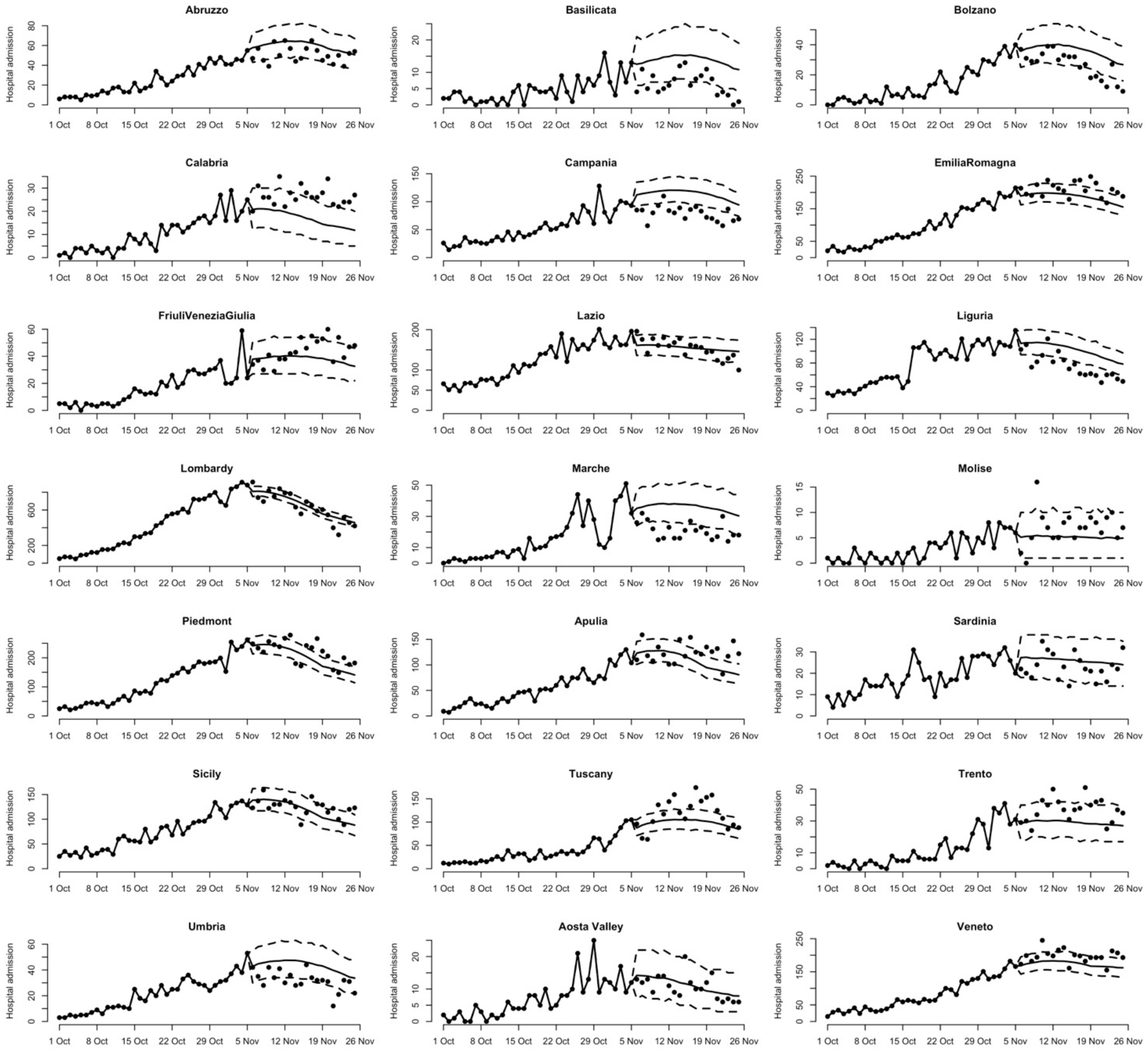

Additional information on the linear mixed models A-E

A bootstrap procedure on Models A-E was used to compute values represented in Figure 3A-B. The net and relative change in transmissibility between the pre-tier period (October 30 – November 5, 2020) and the post-tier period (November 19 – 25, 2020) was defined as NC= Rpost – Rpre and RC= Rpost/Rpre – 1, where Rpre and Rpost were the values of the reproduction numbers in the two periods. For each model, we sampled model residuals with replacement and added them to fitted values of the reproduction numbers to obtain 1000 bootstrapped replicates, used to refit the model and to obtain a distribution of matched values for Rpre and Rpost.

Values reported in Figure 3C were computed for each model using its regression equation and the relevant estimated model parameters (beta). Estimated R values represent the average transmissibility among provinces (model A, B, C, E) or regions (model D) and their 95% confidence interval in the pre- and post-tier period.

2.2 Model selection

We adopted a stepwise model selection procedure based on the likelihood ratio tests to assess if any covariates or interaction terms may be dropped from model A. The performed analysis confirmed that model A should be preferred to all models nested in this formulation. Likelihood ratio tests on the comparison between model A and nested models always resulted in p-values<0.0001 (Table S13)

Table S13 Result of likelihood ratio test comparison between model A and all its nested models (i.e., discarding one covariate at a time). p-values for F-test were computed using Satterthwaite's method considering statistical significance at the 0.05 threshold (p-values <0.05).

Model	Chisq	DF	pvalues
$Y_{p,T} = \beta_0 + \beta_1 X_p^{orange} + \beta_2 X_p^{red} + \beta_3 Z_T$ **(tier+period)**	27.656	2	<0.0001
$Y_{p,T} = \beta_0 + \beta_1 X_p^{orange} + \beta_2 X_p^{red}$ **(tier)**	203.91	3	<0.0001
$Y_{p,T} = \beta_0 + \beta_3 Z_T$ **(period)**	27.904	4	<0.0001

2.3 Sensitivity analyses

We further evaluated the robustness of our results by re-estimating the impact of tiered restrictions on transmissibility with alternative modelling assumptions. We considered the following sensitivity analyses:

1) **Hospital admission.** We used the same model described as model A, but considering as dependent variable $Y_{p,T}$ the reproduction number from hospital admissions, $R^h(t)$, stratified by province and period of observation. Figure S7 shows the temporal change observed in the estimated $R^h(t)$ across different provinces of Italy, before and after the introduction of regional tiered restrictions. Results obtained with this sensitivity analysis are reported in Table S14 and S15, and Figure S8. Table S14 shows that the starting values of $R^h(t)$ (Oct 30 – Nov 5) were more homogeneous across tiers than those of R(t) computed from symptomatic cases. However, estimated reductions associated with different tiers are comparable to those obtained in the main analysis.

Table S14. Result of the linear mixed model on the net reproduction number estimated from hospital admissions, $R^h(t)$, at the provincial level. See Table S2 for interpretation of parameters. p-values for t-statistics were computed using Satterthwaite's method for denominator degrees of freedom considering statistical significance at the 0.05 threshold (p-values <0.05). The estimated marginal and conditional coefficient of determination R^2 computed based on Nakagawa et al. [1] were 0.477 and 0.547, respectively.

PARAMETER	VALUE	STD ERROR	DF	T-VALUE	P-VALUE
β_0	1.393	0.062	39.8	22.478	<0.00001

$\boldsymbol{\beta_1}$	0.067	0.077	37.0	0.873	0.3883
$\boldsymbol{\beta_2}$	0.045	0.077	34.9	0.593	0.5568
$\boldsymbol{\beta_3}$	-0.280	0.067	193.1	-4.191	<0.00001
$\boldsymbol{\beta_4}$	-0.162	0.082	193.1	-1.991	0.0479
$\boldsymbol{\beta_5}$	-0.192	0.080	193.1	-2.399	0.0174

Table S15. Mean net reproduction number estimated from hospital admission, $R^h(t)$, before and after regional interventions.

Maximum tier	Mean Rt^h (95% CI) October 30 – November 5	Mean Rt^h (95% CI) November 19 - 25	Relative reduction (95% CI)
**Yellow**	1.39 (1.27 – 1.52)	1.11 (0.99 – 1.24)	20.1% (11.3 – 28.6%)
**Orange**	1.46 (1.37 – 1.55)	1.02 (0.93 – 1.11)	30.3% (24.6 – 35.6%)
**Red**	1.44 (1.35 – 1.53)	0.97 (0.88 – 1.05)	32.8% (27.5 – 38.3%)

Figure S7. Temporal dynamics of the net reproduction numbers from hospital admissions, $R^h(t)$, and of restrictions applied between October 30 and November 25. Each line shows the mean $R^h(t)$ for an Italian province (grey) or region (blue). Provinces are grouped by region as interventions were carried out at the regional level. Colored rectangles refer to the timeframe when the different tiers were in place (see Table 1 in main text for a description of restrictions).

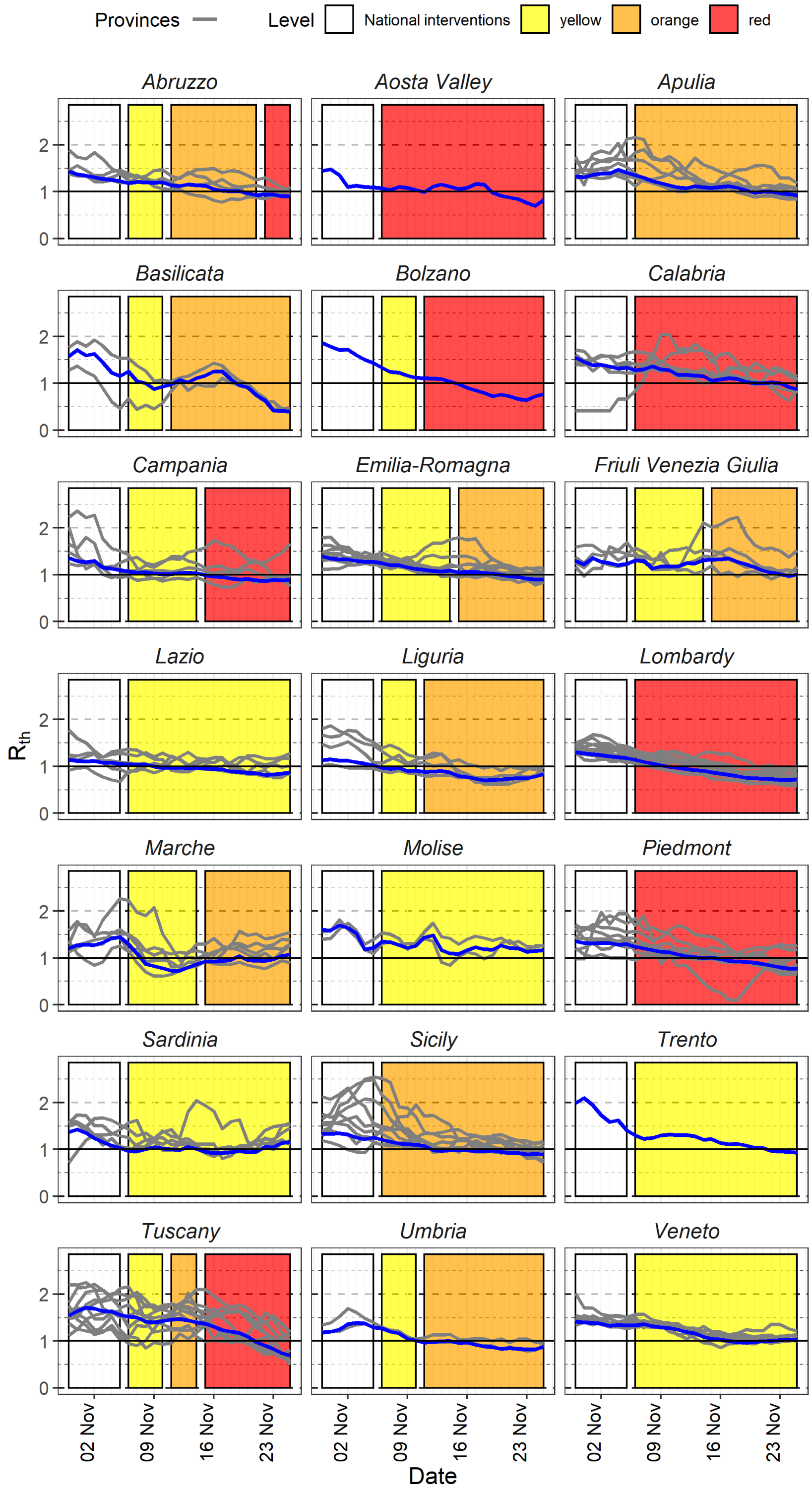

.

Figure S8 Variation in the net reproduction number from hospital admissions ($R^h(t)$) in each province. The arrows indicate the variation in $R^h(t)$ from the period before the introduction of the regional tiered restrictions (October 30-November 5) to the end of our observations (November 19 – 25). Provinces are ordered by decreasing reduction in $R^h(t)$.

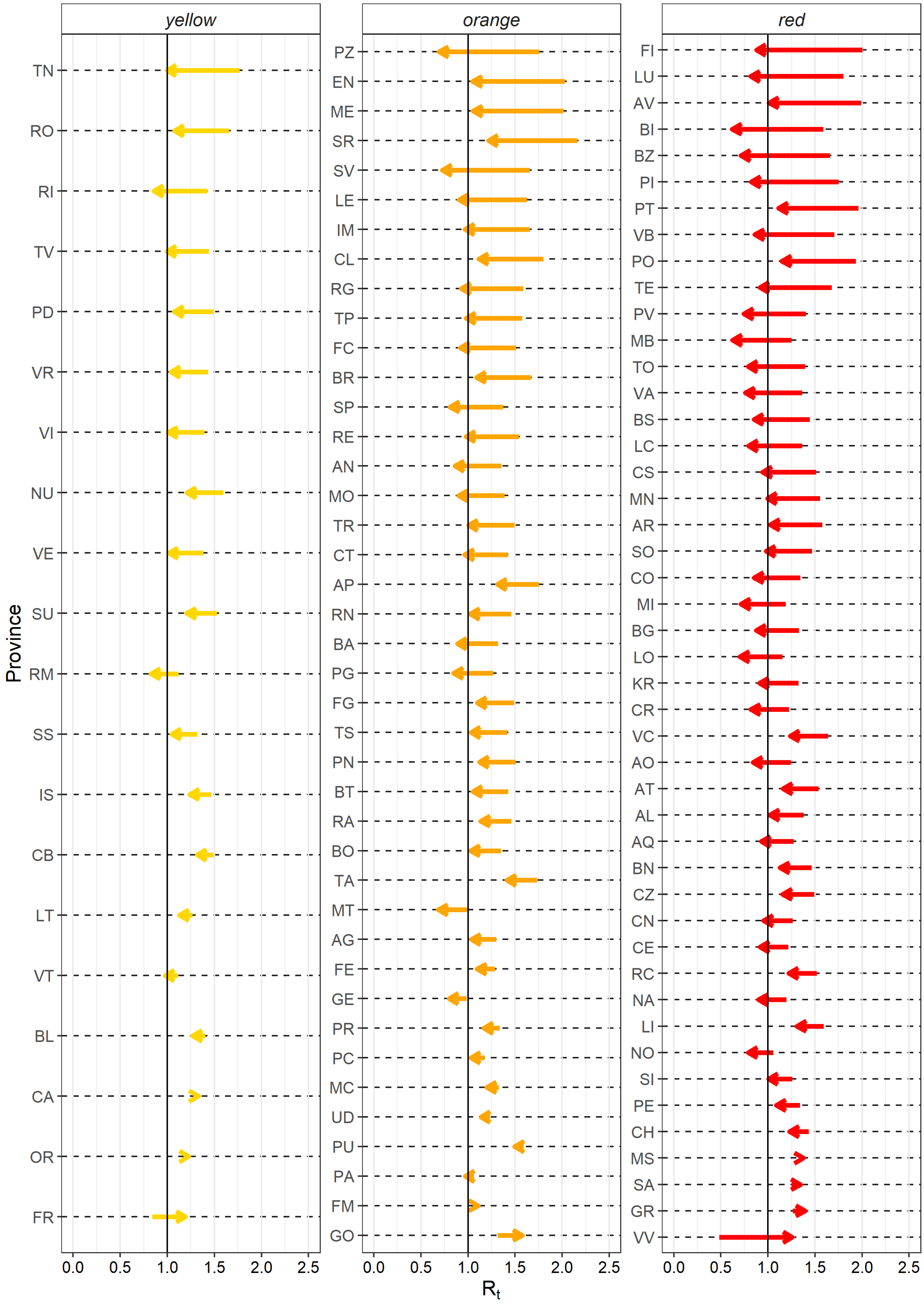

2) **Lengths of periods T.** We applied model A to R(t) values computed at provincial level but considering average values over alternative period lengths ranging from 3 to 11 days. For example, when considering a period length of 3 days, we compared the mean R(t) in period November 3 – 5 against November 23-25. Figure S9 shows the difference between model parameters estimated for each period length compared to the main analysis (7 days). Estimated variations in parameter estimates are limited (within 0.05 in most cases).

Figure S9 Variation in estimated model parameters when considering different lengths (in days) of the window over which R(t) is averaged. On the x axis the difference between the estimated parameter value of the parameter and the main analysis (which uses a 7-day window). Parameter names are on the y axis. See Table S2 for interpretation of parameters. Colors represent different period lengths. Asterisks represent statistical significance at the 0.05 threshold (p-values <0.05) of the estimated parameters from a linear mixed model (see Model A for further details, p-values for t-statistics were computed using Satterthwaite's method for denominator degrees of freedom).

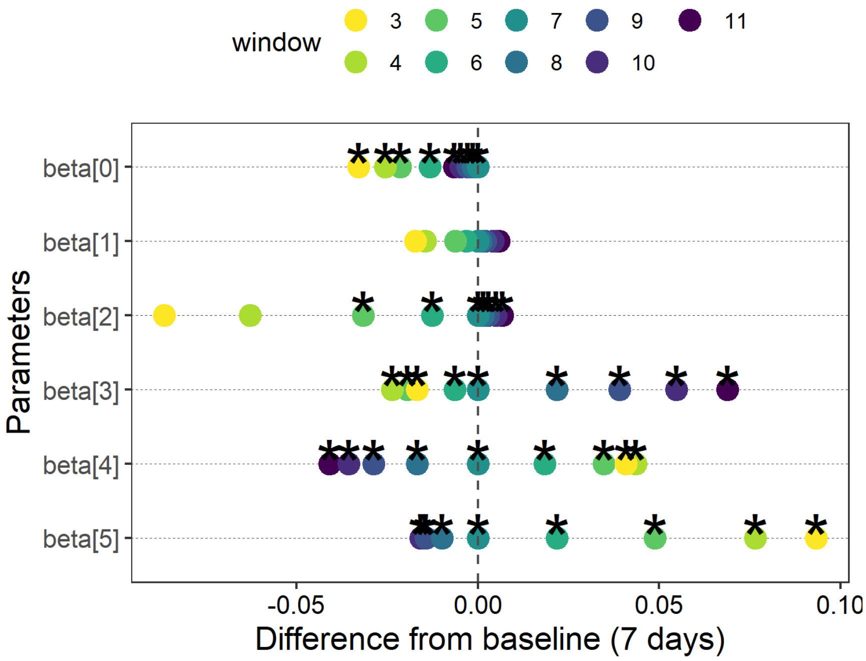

Figure S10 Estimates from LMMs using different time windows. A) Net change of the reproduction number between the period pre tier restrictions, and the period post tier restrictions, by tier group, n=107 observations (provinces) observed pre/post tiers. B) Relative change of the reproduction number between the period pre tier restrictions, and the period post tier restrictions, by tier group, n=107 observations (provinces) observed pre/post tiers. C) Mean reproduction number in the period pre tier restrictions, and the period post tier restrictions, by tier group, n=107 observations (provinces) observed pre/post tiers. Dots (centre of the error bars) represent the mean values, vertical lines represent 95%CI.

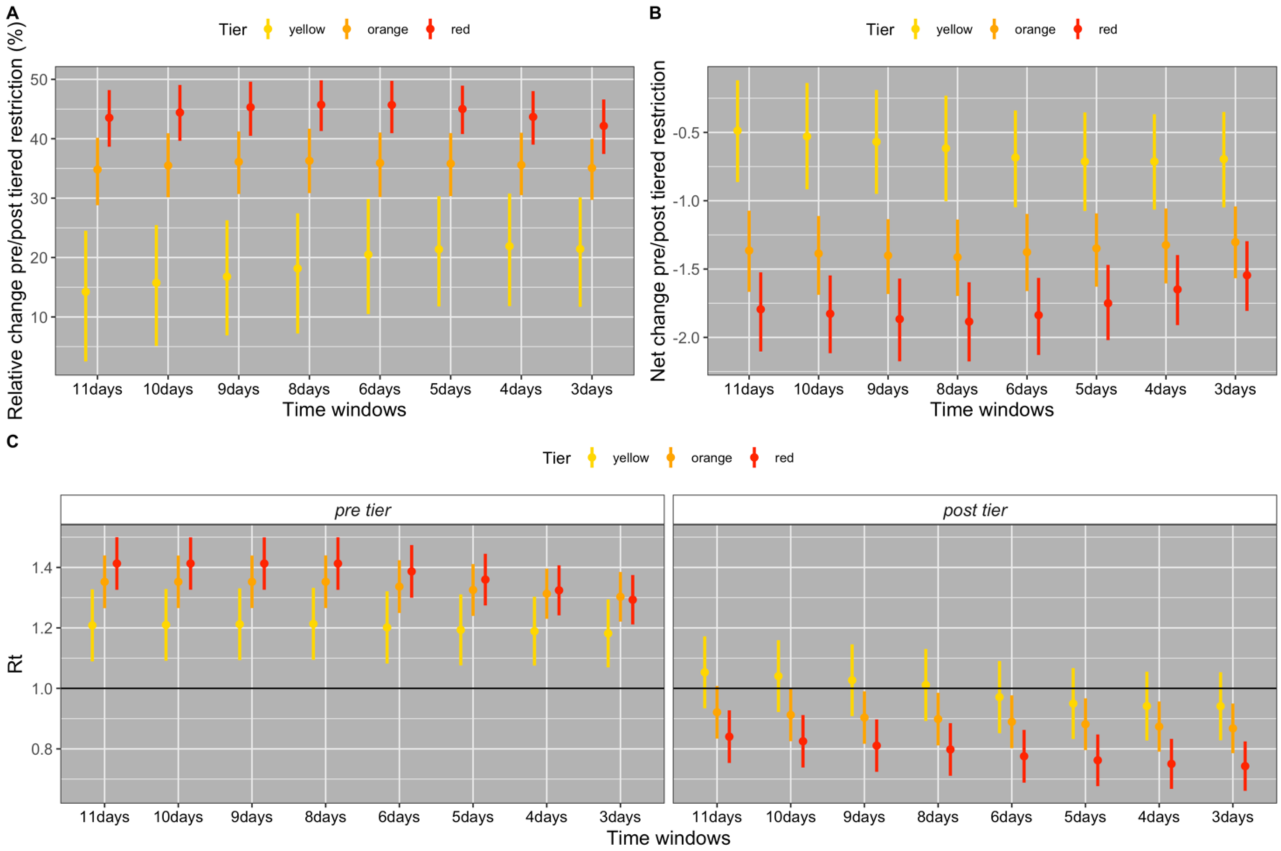

3) **Alternative grouping of provinces.** We applied a model that is a hybrid between model A and model C, where we categorized tiers in five groups L1-L5. Groups L1, L3 and L5 correspond to the grouping in model C, i.e., provinces that never changed tiers; Groups L2 and L4 contain provinces that changed tiers, by maximum assigned tier (orange for L2, red for L4). Please refer to Figure 2 in the main text for a reference of category assignments:

- L1: tier constantly yellow (20 provinces): Lazio (5 provinces), Molise (2 provinces), Sardinia (5 provinces), Trento (1 Autonomous Province), Veneto (7 provinces);
- L2: tier reaching up to orange (26 provinces): Basilicata (2 provinces), Emilia-Romagna (9 provinces), Friuli Venezia Giulia (4 provinces), Liguria (4 provinces), Marche (5 provinces), Umbria (2 provinces);
- L3: tier constantly orange (15 provinces): Apulia (6 provinces), Sicily (9 provinces)
- L4: tier reaching up to red (20 provinces): Abruzzo (4 provinces), Bolzano (1 Autonomous Province), Campania (5 provinces), Tuscany (10 provinces);
- L5: tier constantly red (26 provinces): Aosta Valley (1 province), Calabria (5 provinces), Lombardy (12 provinces), Piedmont (8 provinces).

The model equation is as follows

$$Y_{p,T} = \beta_0 + \beta_1 X_p^{L2} + \beta_2 X_p^{L3} + \beta_3 X_p^{L4} + \beta_4 X_p^{L5} + \beta_5 Z_T + \beta_6 X_p^{L2} Z_T + \beta_7 X_p^{L3} Z_T + \beta_8 X_p^{L4} Z_T + \beta_9 X_p^{L5} Z_T + a_r + b_{r,p} + \varepsilon_{p,T}$$

where

- $Y_{p,T}$ represents the mean value of R(t) in each of the 107 Italian provinces (*p*), averaged over two possible time periods (*T*): October 30 to November 5 (i.e., when nationwide interventions were still in place) or November 19 to November 25 (i.e., two to three weeks after the introduction of the tier system).
- X_p^l is a binary variable set to 1 if province *p* belongs to a region assigned to group l (L1 to L5, see above), and 0 otherwise;
- Z_T is a binary variable set to 0 if T=October 30 – November 5 and to 1 if T=November 19 – November 25;
- $\beta_0, \beta_1, \beta_2, \beta_3, \beta_4, \beta_5, \beta_6, \beta_7, \beta_8$ and β_9 are model parameters, with β_0 representing the average value of R(t) during the period October 30 – November 5 for provinces with maximum tier yellow;
- a_r and $b_{r,p}$ are random effects, assumed to be normally distributed: a_r allows random deviations from the mean R(t) among regions, $b_{r,p}$ allows random deviations from the regional mean R(t) among provinces within a region;
- $\varepsilon_{p,T}$ is random noise assumed to be normally distributed.

The obtained results were substantially equivalent to those presented in the main text (see Table S16 and S17).

Table S16 Result of the linear mixed model on the net reproduction number estimated from symptom onset R(t) at provincial level considering 5 groups of interventions. p-values for t-statistics were computed using Satterthwaite's method for denominator degrees of freedom considering statistical significance at the 0.05 threshold (p-values <0.05). The estimated marginal and conditional coefficient of determination R^2 computed based on Nakagawa et al. [1] were 0.618 and 0.658, respectively

PARAMETER	INTERPRETATION	VALUE	STD ERROR	DF	T-VALUE	P-VALUE
$\boldsymbol{\beta_0}$	Mean R(t) before interventions for provinces in regions L1	1.215	0.055	35.1	22.001	<0.00001
$\boldsymbol{\beta_1}$	Difference in the mean R(t) before interventions for provinces in regions L2, compared to L1	0.103	0.074	34.8	1.404	0.16908
$\boldsymbol{\beta_2}$	Difference in the mean R(t) before interventions for provinces in regions L3, compared to L1	0.207	0.089	23.7	2.319	0.02933
$\boldsymbol{\beta_3}$	Difference in the mean R(t) before interventions for provinces in regions L4, compared to L1	0.345	0.080	30.6	4.304	0.00016
$\boldsymbol{\beta_4}$	Difference in the mean R(t) before interventions for provinces in regions L5, compared to L1	0.071	0.077	26.5	0.921	0.36516
$\boldsymbol{\beta_5}$	Reduction in R(t) after interventions for provinces in regions L1	-0.224	0.062	190.5	-3.598	0.00041
$\boldsymbol{\beta_6}$	Additional reduction in R(t) after interventions for provinces in regions L2, on top of reduction afforded by L1	-0.199	0.083	190.5	-2.401	0.01733
$\boldsymbol{\beta_7}$	Additional reduction in R(t) after interventions for provinces in regions L3, on top of reduction afforded by L1	-0.299	0.095	190.5	-3.133	0.00200
$\boldsymbol{\beta_8}$	Additional reduction in R(t) after interventions for provinces in regions L4, on top of reduction afforded by L1	-0.512	0.088	190.5	-5.820	<0.00001
$\boldsymbol{\beta_9}$	Additional reduction in R(t) after interventions for provinces in regions L5, on top of reduction afforded by L1	-0.318	0.083	190.5	-3.842	0.00017

Table S17. Mean net reproduction number estimated from symptom onset, Rt, before and after regional interventions.

Tier	Mean Rt (95% CI) October 30 – November 5	Mean Rt (95% CI) November 19 - 25	Relative reduction (95% CI)
**L1**	1.21 (1.11 – 1.32)	0.99 (0.88 – 1.10)	18.2% (8.8 – 27.1%)
**L2**	1.32 (1.22 – 1.41)	0.90 (0.80 – 0.99)	32.1% (21.4 – 39.0%)
**L3**	1.42 (1.28 – 1.56)	0.90 (0.76 – 1.04)	36.8% (27.6 – 44.6%)
**L4**	1.56 (1.45 – 1.67)	0.82 (0.71 – 0.94)	47.3% (40.8 – 53.6%)
**L5**	1.29 (1.18 – 1.39)	0.74 (0.64 – 0.85)	42.2% (35.4 – 49.2%)

References

1. Nakagawa S, Johnson PC, Schielzeth H. The coefficient of determination R2 and intra-class correlation coefficient from generalized linear mixed-effects models revisited and expanded. Journal of the Royal Society Interface, 2017; 14(134), 20170213.
2. Ali ST, Wang L, Lau EHY, Xu XK, Du Z, Wu Y, Leung GM, Cowling BJ, Serial interval of SARS-CoV-2 was shortened over time by nonpharmaceutical interventions. Science 2020; 369 (6507), 1106-1109
3. Marziano V, Guzzetta G, Mammone A, Riccardo F, Poletti P, Trentini F et al. Return to normal: COVID-19 vaccination under mitigation measures. medRxiv 2021. Accessible at https://www.medrxiv.org/content/10.1101/2021.03.19.21253893v1
4. Wallinga J and Lipsitch M. How generation intervals shape the relationship between growth rates and reproductive numbers. Proceedings of the Royal Society B: Biological Sciences, 2007, 274.1609: 599-604.
